# A comprehensive multi-omics and functional study of evolutionary adaptive responses to smoke

**DOI:** 10.1016/j.isci.2026.115547

**Published:** 2026-04-01

**Authors:** Simon D. Pouwels, Hao Chen, Senani N.H. Rathnayake, Andy Lan, Rashad M. Mahbub, Anna Chi Ying Yeung, Corry-Anke Brandsma, Thamar J. Lobo, Victor Guryev, Irene H. Heijink, Maarten van den Berge, Alen Faiz

**Affiliations:** 1 University of Groningen, University Medical Center Groningen, Department of Pathology and Medical Biology, Groningen, the Netherlands; 2 University of Groningen, University Medical Center Groningen, Groningen Research Institute for Asthma and COPD, Groningen, the Netherlands; 3 University of Groningen, University Medical Center Groningen, Department of Pulmonary Diseases, Groningen, the Netherlands; 4 Centre for Inflammation, Centenary Institute and University of Technology Sydney, School of Life Sciences, Sydney, NSW, Australia; 5 University of Technology Sydney, Respiratory Bioinformatics and Molecular Biology (RBMB), School of Life Sciences, Sydney, NSW, Australia; 6 European Research Institute for the Biology of Ageing, University of Groningen, University Medical Center Groningen, Groningen, the Netherlands

**Keywords:** Toxicology, Bioinformatics, Complex system biology

## Abstract

Cigarette smoking remains one of the leading causes of preventable death worldwide. Upon inhalation, smoke induces damage to the lung epithelium, which can be the driving force for the development of chronic lung diseases. In the current study, we identified the “smoking signature” by assessing transcriptomic differences between current and non-smokers in multiple cohorts. By mapping the smoking signature onto single-cell and spatial transcriptomics data, we identified the surface layer of the airway epithelium as the part of the airways most affected by smoke. Next, by comparing the smoking signature between lungs of humans and non-human primates, we identified a cluster of human-specific smoke-response genes, suggesting an evolutionary adaptation to smoke exposure. Methylation and ChIP-seq analyses were used to identify *NRF2* and *AhR* as master regulators of the smoking signature. Knockout of smoking signature-genes *NQO1* and *ALDH3A1* revealed their role in protective responses of the airways upon smoke exposure.

## Highlights

• The smoking signature distinguishes current from non-smoking subjects
• Cigarette smoke mainly affects the surface layer of the airway epithelium
• Humans have evolutionary adapted to the inhalation of smoke
• The expression of *NQO1* and *ALDH3A1* protects against the damaging effects of smoke

## Graphical abstract

**Figure:**
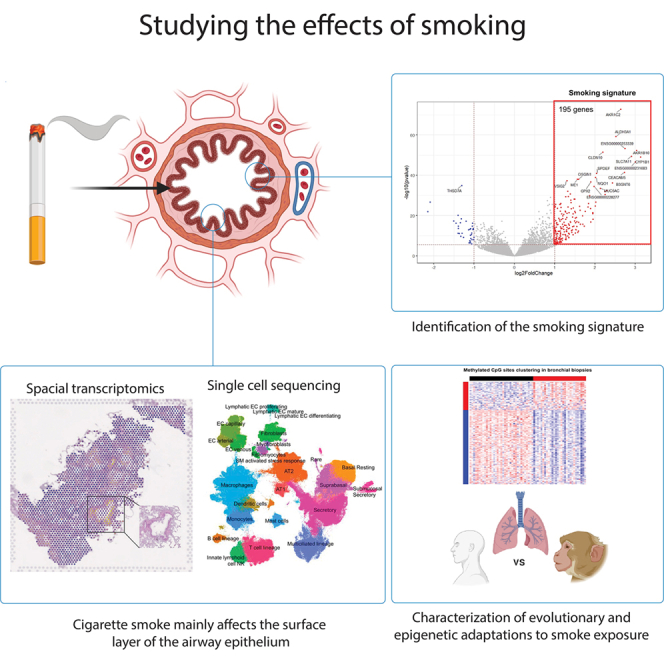

## Introduction

Cigarette smoking remains one of the leading causes of preventable death worldwide. It is a well-known risk factor for the development of various diseases, including lung cancer, cardiovascular disease, and chronic obstructive pulmonary disease (COPD). Smoking, whether acute or chronic, profoundly impacts gene expression throughout the airways.^1^^,^^2^^,^^3^ The airway epithelium provides the first line of physical defense to protect submucosal layers from inhaled toxicants, such as cigarette smoke, to minimize cellular damage.^4^^,^^5^

Airway epithelium acts as a chemical barrier by releasing antioxidants, antiproteases, antimicrobial peptides, and bioactive cytokines, thereby initiating innate immune responses and safeguarding the airways against foreign toxicants and pathogens.^4^^,^^5^ Airway lining comprises a complex structure featuring various specialized cell types such as ciliated cells, secretory (club and goblet) cells, and basal cells within a ciliated pseudostratified columnar and differentiated epithelial layer, as well as rare epithelial cells such as tuft cells, pulmonary ionocytes, and pulmonary neuroendocrine cells.^4^ Among these cell types, basal cells, located in the inner layer of the airway epithelium, serve as key progenitors that differentiate into secretory and ciliated cells in the airway surface epithelial layer.

Previous findings have demonstrated that cigarette smoke impairs the airway epithelial barrier function.^6^ We postulate that with the evolution of humans and the advent of cooking with fire, exposure to smoke has been a consistent aspect of human history, potentially driving adaptations in the human airway to counteract the detrimental effects of smoke exposure. Over generations, prolonged exposure to smoke may have induced epigenetic modifications, such as changes in DNA methylation patterns or histone modifications that influenced the expression of genes critical for adaptive responses to environmental toxins.^7^ These modifications could represent a form of evolutionary plasticity, where the human genome has responded to repeated smoke exposure through regulatory changes that enhance protection against lung damage. Here, we hypothesize that epigenetic changes induce increased expression of genes associated with oxidative stress and detoxification responses, thereby offering adaptive protection against lung damage in human smokers. Specifically, these DNA methylation sites likely reside within or near the binding sites of transcription factors (TFs) sensitive to cigarette smoke, enabling direct regulation of key genes that mediate protein-level changes in response to smoke exposure.

Most existing studies used the airway epithelium of the upper respiratory tract (trachea)^8^ or *in vitro*-cultured airway epithelial cells,^9^ overlooking the direct effect of smoke exposure on the airways within the lungs and the impact across multiple layers of omics. To address this, we employed a comprehensive multi-omics approach on respiratory samples exposed to smoke, comparing the transcriptomic differences between human and non-human primate lungs, to highlight the evolutionary adaptations of the human airways to withstand smoke exposure.

The first aim of this study was to identify a “smoking signature” gene set that can distinguish current smokers from never smokers across multiple cohorts. Furthermore, we aimed to identify whether the smoking signature is a human-specific evolutionary adaptation to smoke. Additionally, we aimed to identify the exact cell-types and cellular structures affected by smoke in the human airways. Lastly, we aimed to identify the TFs as well as genes regulating TF expression, associated with the adaptive responses to smoke exposure.

## Results

### Generation and evaluation of the smoking signature

**Figure 1:**
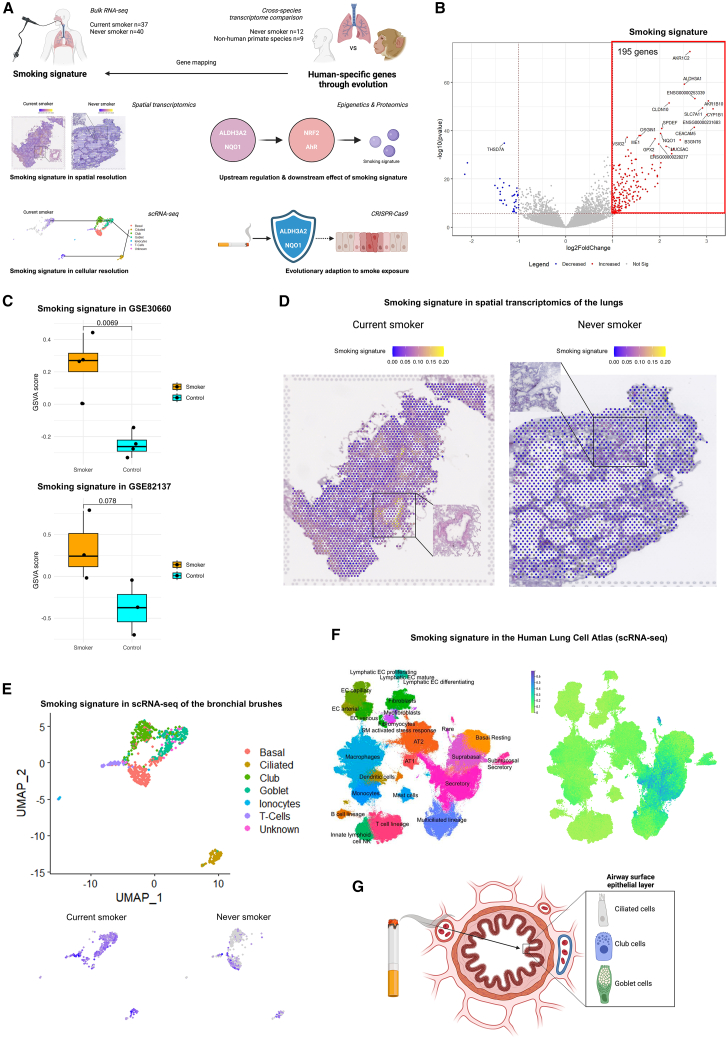
Generation and evaluation of the smoking signature at the transcriptome level (A) Schematic representation of the experimental design of the study. (B) Volcano plot of significantly differentially expressed genes in bronchial biopsies from current smokers (*n* = 37) compared to never smokers (*n* = 40) within the NORM cohort (ClinicalTrials accession no.: NCT00848406). A total of 195 significantly upregulated genes, highlighted within the boxed area, constitute the “smoking signature.” Significance was determined by a log2 fold change >1 and a Benjamini-Hochberg (BH) adjusted *p* value <0.05. (C) Boxplot of gene set variation analysis (GSVA) signaling scores of the smoking signature in two independent datasets of *in vitro* smoke-exposed airway epithelial cells cultured at air-liquid interface (ALI).^10^^,^^11^ The center line corresponds to the median, the top and bottom hinges delineate the first and third quartiles, respectively. (D) Mapping score of the smoking signature in 10× Visium spatial transcriptomics dataset^12^ of lung regions from a current smoker and a never smoker, predominantly consisting of alveoli with minimal airway presence. The boxed area highlights the airway region. (E) Uniform manifold approximation and projection (UMAP) of a single-cell RNA sequencing (scRNA-seq) dataset of bronchial brushes of current smokers (*n* = 6) and never smokers (*n* = 6)^13^ (top), and mapping score of the smoking signature based on the smoking status (bottom). (F) UMAP of the Human Lung Cell Atlas (left) and the mapping scores of the smoking signature (right). (G) Illustration of the main effect of cigarette smoke on the airway surface epithelial layer.

To precisely define asymptomatic smokers, bulk RNA sequencing (RNA-seq) was applied on bronchial biopsies of asymptomatic current smokers (*n* = 37) and never smokers (*n* = 40) from the normal values of inflammatory variables from healthy subjects (NORM) cohort. This analysis revealed a small group of 35 significantly downregulated genes and a large group of 195 significantly upregulated genes in response to cigarette smoking. The group of upregulated genes together we referred to as the “smoking signature” (Figure 1B and Table S2). This signature includes genes related to detoxification, mucus production, and cancer (log2 fold change >1, adjusted *p* < 0.05, Table S3), consistent with known pathogenic effects of cigarette smoking. The robustness of the smoking signature was confirmed by its upregulation in two independent datasets of *in vitro* smoke-exposed airway epithelial cells cultured at air-liquid interface (ALI)^10^^,^^11^ (Figure 1C).

Table S2. Smoking signature

To verify the specificity of the smoking signature to the airways, it was mapped onto a publicly available 10× Visium spatial transcriptomics dataset of age- and sex-matched asymptomatic current smoker (*n* = 1) and never smoker (*n* = 1) lungs.^12^ The smoking signature was enriched in the airways of current smokers, even in a lung region predominantly consisting of alveoli, showing higher expression of the smoking signature compared to never smokers (Figure 1D). For greater cellular resolution, single-cell RNA sequencing (scRNA-seq) data were used from bronchial brushes of current smokers (*n* = 6) and never smokers (*n* = 6).^13^ The smoking signature was primarily expressed by goblet/club cells and ciliated cells in bronchial brush samples (Figure 1E), with minimal changes observed in basal cells, indicating that the direct response to smoke mainly occurs in the surface layer of the airway epithelium, which is directly exposed to inhaled smoke. Next, this observation was validated using the Human Lung Cell Atlas,^14^ confirming the predominant expression of the smoking signature in goblet, club, and ciliated cells, while basal cells showed no prominent alterations (Figure 1F). These findings underscore the critical role of the surface layer of the airway epithelium in response to cigarette smoke (Figure 1G).

### Evolutionary adaptation to smoke in human airways

Over generations, prolonged exposure to smoke may have induced epigenetic modifications, such as changes in DNA methylation patterns that influenced the expression of genes critical for adaptive responses to environmental toxins.^7^ These modifications could represent a form of evolutionary plasticity, where the human genome has adapted to repeated smoke exposure through regulatory changes that enhance protection against lung damage. Examples of such adaptive plasticity exist in other human populations. For instance, the Bajau sea nomads display enlarged spleens and genetic variants in PDE10A that facilitate prolonged deep-sea diving, illustrating how repeated environmental pressures can drive evolutionary adaptation in regulatory pathways.^15^

**Figure 2:**
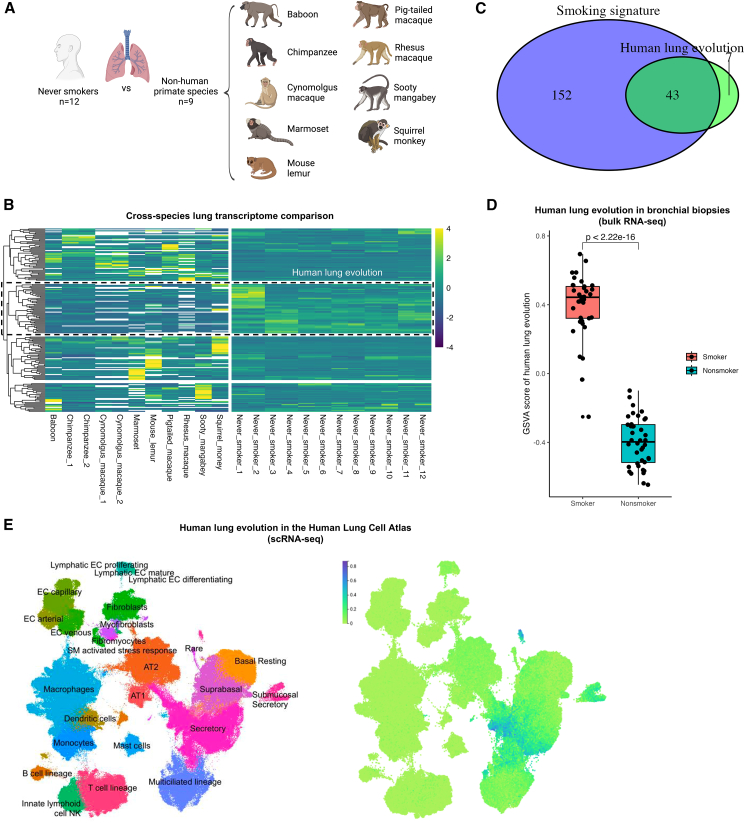
Evolutionary adaptation to cigarette smoke exposure in human airways (A) Illustration of cross-species lung transcriptome analysis comparing human never smokers (*n* = 12) and non-human primate species (*n* = 9 species). (B) Heatmap of a total of four clusters containing significantly differentially expressed genes between the lungs of human never smokers and non-human primates. The boxed cluster, with higher expression in humans, represents the “human lung evolution” signature. (C) Venn diagram showing the overlap between the “human lung evolution” signature and the smoking signature. (D) Boxplot of GSVA signaling scores of the “human lung evolution” signature in the bulk RNA-seq of bronchial biopsies collected from current smokers and never smokers from the NORM cohort. Each scatter represents one participant. The center line corresponds to the median, the top and bottom hinges delineate the first and third quartiles, respectively. (E) UMAP of the Human Lung Cell Atlas (left) and the mapping scores of the “human lung evolution” signature (right).

Since the use of fire for cooking is a relatively recent development in human evolution, we sought to determine whether this protective mechanism is unique to humans or conserved across primate species. To address this, the lung transcriptomes of humans were compared to those of other primates, including chimpanzee, baboon, cynomolgus macaque, marmoset, pig-tailed macaque, rhesus macaque, sooty mangabey, and squirrel monkey, all of which were collected from the non-human primate reference transcriptome resource^16^ (Figure 2A). Cross-species transcriptomic comparison identified four distinct clusters of genes with lineage-specific upregulation (Figure 2B). Of which, a cluster (named “human lung evolution”) showed significantly higher expression in human lungs compared to non-human primates. Interestingly, a large overlap between the human lung evolutionary genes and the smoking signature was found. (Figure 2C) Furthermore, similarly high expression of human lung evolutionary genes was found in bronchial biopsies of human smokers (Figure 2D), which evidenced that evolution plays a role in the response to smoke in humans. Mapping of human lung evolutionary genes onto the Human Lung Cell Atlas^14^ revealed predominant enrichment in goblet, club, and ciliated cells (Figure 2E), all of which exactly overlap with the cell types affected by the smoking signature, suggesting that the key evolutionary differences between human and non-human primate lungs lie in the same surface layer of the airway epithelium that directly interacts with smoke.

### NRF2 and AhR as master regulators of the smoking signature

**Figure 3:**
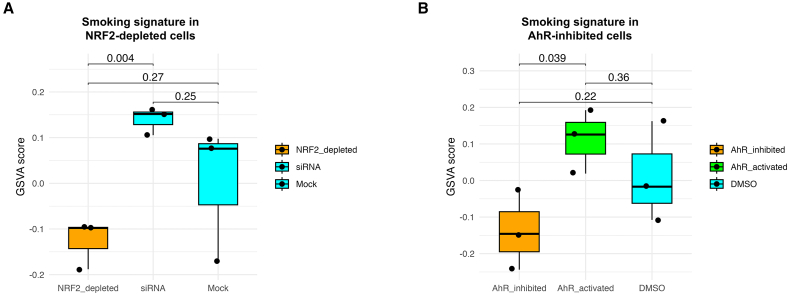
NRF2 and AhR as master regulators of the smoking signature (A and B) Boxplots of GSVA signaling scores of the smoking signature in NRF2-depleted cells (A) and in AhR-inhibited cells (B). The center line corresponds to the median, the top and bottom hinges delineate the first and third quartiles, respectively.

Next, it was investigated whether these human-specific genes were regulated by the same epigenetic mechanisms controlling gene expression of the smoking signature. Among the genes in the smoking signature, there are two key TFs, NRF2 and AhR. Suppression of NRF2^10^ (NCBI-GEO: GSE38332) and AhR^11^ (NCBI-GEO: GSE109576) in A549 human alveolar basal epithelial cells led to a decreased expression of the smoking signature upon smoke exposure, supporting their role as master regulators of the smoking signature (Figures 3A and 3B). Subsequently, it was investigated whether the genomic proximity of NRF2 and AhR contained binding sites of other genes of the smoking signature using chromatin immunoprecipitation sequencing (ChIP-seq) analysis prior- and post-activation with D,L-sulforaphane16 and 2,3,7,8-tetrachlorodibenzo-p-dioxin,^17^ respectively, in NRF2- and AhR-inhibited A549 cells. Initially, genes of the smoking signature that were downregulated during AhR suppression were investigated (Table S4). In AhR-inhibited cells, 60.0% of the suppressed genes of the smoking signature exhibited an AhR binding peak within a 100 KB region flanking their transcription start site (TSS), indicating direct binding of the majority of AhR-altered genes by the protein. Next, in NRF2-inhibited cells, only 21.9% of the suppressed genes of the smoking signature (Table S4) showed an NRF2 binding peak within a 100 KB region flanking their TSS.

Table S4. Differential ChIP-seq analysis of smoking signature

While these ChIP-seq datasets were generated using chemical activators, rather than cigarette smoke, they serve as valuable resources for identifying high-confidence binding sites. Although the activation strength may differ, these reference datasets were used to complement the functional experiments, which directly demonstrated the role of NRF2 and AhR in regulating smoking-responsive genes during smoke exposure in epithelial cells.

### ALDH3A1 and NQO1 as upstream epigenetic regulators of the smoking signature

**Figure 4:**
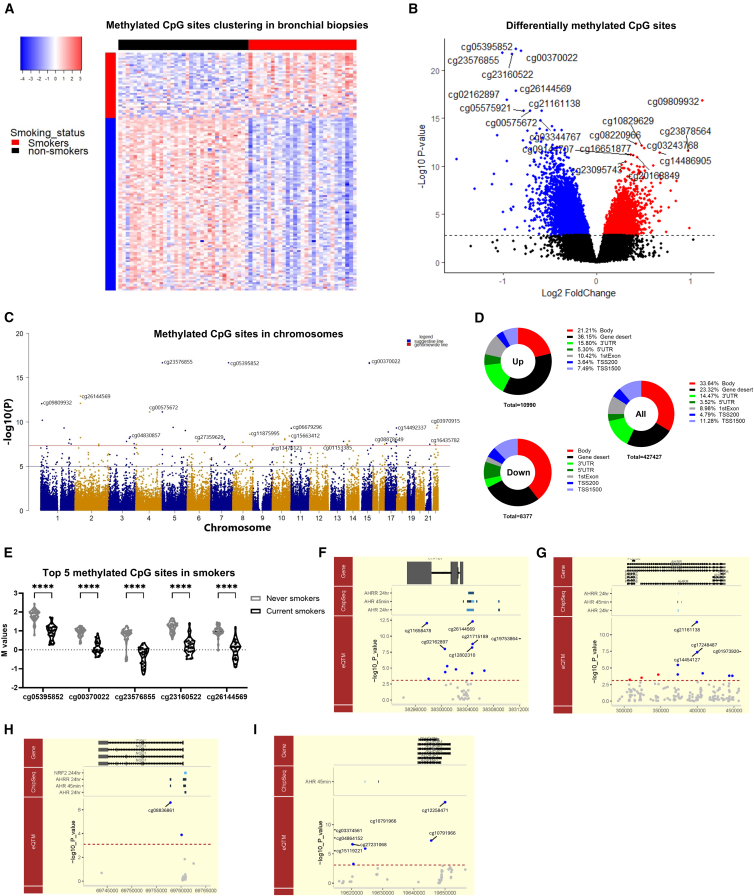
ALDH3A1 and NQO1 as upstream epigenetic regulators of the smoking signature Heatmap (A) and volcano plot (B) of the significantly differentially methylated CpG sites in the bronchial biopsies collected from current smokers (*n* = 33) and never smokers (*n* = 36) from the NORM cohort. BH-adjusted *p* value <0.05. (C) Manhattan plot of the differentially methylated CpG sites between current and never smokers and their positions on chromosomes. (D) Positional methylated CpG sites upon smoke exposure. (E) Violin plot of the top 5 significantly methylated CpG sites. Data shown as individual data points, ∗∗∗∗*p* < 0.0001. (F–I) Expression quantitative trait methylation (eQTM) analysis in relation to transcription factor binding regions for CYP1B1 (F), ALDH3A1 (G), AhRR (H), and NQO1 (I). Genes located in the identified genomic region are shown in the top image, the black arrows indicate the direction of transcription. The middle image shows the binding of transcription factors by ChIP-seq analysis. Bottom image demonstrates the eQTM, the –log10 *p* value (*y* axis) against genomic location (*x* axis). eQTMs with a false discovery rate (FDR) < 0.05 and with a negative association with gene expression are represented as blue points, while positive associations are represented as red points. All other eQTMs that were not found to be significant are depicted as gray points.

Initially, DNA methylation profiling of bronchial biopsies from asymptomatic current (*n* = 33) and never smokers (*n* = 36) revealed 14,186 differentially methylated CpG sites, well-differentiated between smokers and non-smokers (Figures 4A–4C). Notably, CpG methylation was over-represented in the 5′ untranslated (UTR) and gene body regions (Figure 4D), suggesting direct regulation of gene expression by smoking-induced DNA methylation given their position in the promotor region. Key CpG sites include cg00370022, upstream of the detoxification gene *CYP1A1*, and cg23576855 in AhR repressor (*AhRR*), both involved in the AhR signaling pathway (Figure 4E and Table S5).

Table S5. Top 50 CpG sites differentially methylated between the bronchial biopsies collected from asymptomatic current smokers and never smokers

To bridge the changes of DNA methylation with the regulation of gene expression of the smoking signature, *cis*-expression quantitative trait methylation (eQTM) analysis (1 MB flanking the gene) was performed using bronchial biopsies from asymptomatic current (*n* = 29) and never smokers (*n* = 35). Among 1,882 identified eQTMs, 143 (7.6%) were located within the binding sites of TFs, including NRF2, AhR, and AhRR. Notably, the *CYP1B1* gene showed the strongest eQTM, located upstream of the TSS of *CYP1B1* and within the TF binding sites of both *AhR* and *AhRR* (Figure 4F). Similarly, eQTMs for the *ALDH3A1* gene were found within AhR binding sites and positioned 25,000 bp downstream of its TSS (Figure 4G). Additionally, eQTM for the *AhRR* gene was situated within the TF binding sites of *AhR* and *AhRR*, positioned 50,000 bp downstream to the TSS of the *ALDH3A1* gene (Figure 4H). Moreover, eQTMs for the *NQO1* gene were located 2,500 bp downstream to the TSS of the *NRF2* gene (Figure 4I).

### ALDH3A1 and NQO1 as guardians of epithelial survival and barrier function upon smoke exposure

**Figure 5:**
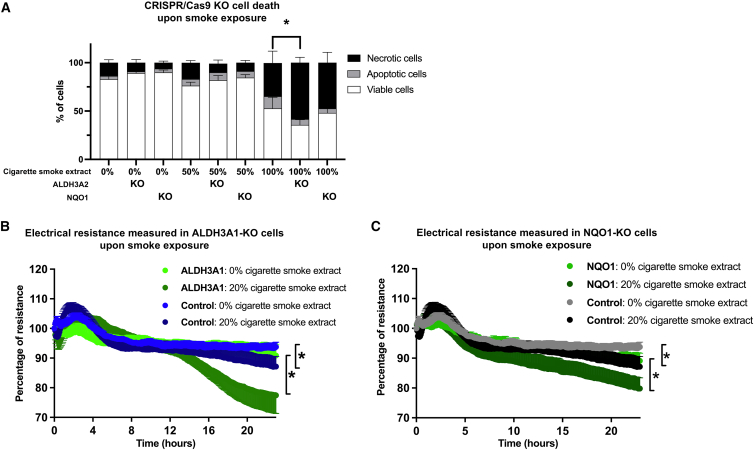
ALDH3A1 and NQO1 as guardians of epithelial survival and barrier function upon smoke exposure (A) A549 epithelial cell death upon 4 h of exposure to 0%–100% cigarette smoke extract in ALDH3A1 and NQO1 CRISPR-Cas9 knockout cells. Epithelial cell barrier function change in response to 0%–20% cigarette smoke extract exposure for 24 h, which was measured in real-time monitoring of electrical resistance and capacitance during and upon the establishment of epithelial monolayers using electric cell-substrate impedance sensing (ECIS) in ALDH3A1- (B) and NQO1-knockout (C) A549 cells. ∗ *p* value <0.05.

ALDH3A1 and NQO1 are among the top differentially expressed genes of our smoking signature (Table S1). Likewise, previous studies have shown upregulation of genes related to aldehyde and ketone metabolism, including *ALDH3A1* and *NQO1* in whole lungs^18^ and in airway epithelial cells upon smoking.^19^^,^^20^ To investigate their role in cellular functions such as survival and epithelial barrier integrity upon smoke exposure, CRISPR-Cas9 knockout (KO) cell lines were generated using A549 human alveolar basal epithelial cells. Cell death was assessed by measuring the number of necrotic and apoptotic cells in CRISPR-Cas9 KO A549 cells after 4 h of cigarette smoke extract (CSE) exposure, followed by a 24 h CSE-free incubation period. Without exposure to CSE, the percentage of viable, apoptotic or necrotic cells is similar between wild type A549 cells and ALDH3A1 or NQO1 KO cells (Figure 5A). Similarly, upon exposure to 50% CSE, no differences in susceptibility for CSE-induced cell death was observed between wild type and KO cells. However, upon 100% CSE exposure, ALDH3A1 KO cells displayed significantly higher levels of necrotic cells compared to the wild type A549 or NQO1 KO cells. This suggests that the downregulation of ALDH3A1 increased the susceptibility for CSE-induced necrosis upon extreme exposure only.

Next, the barrier function in response to smoke exposure was measured in real-time by monitoring electrical resistance and capacitance during and after the establishment of epithelial monolayers using electric cell-substrate impedance sensing (ECIS). The cells were stimulated to 20% CSE to avoid the induction of cell death. Although the baseline barrier integrity was similar, wild type cells exposed to 20% CSE experienced reduced monolayer integrity, which was further compromised in ALDH3A1 and NQO1 KO cells (Figures 5B and 5C). In contrast, no significant differences were observed in CYP1A1 and CYP1B1 KO cell lines. These findings suggest that ALDH3A1 plays a protective role, as its KO leads to increased necrosis and epithelial barrier disruption upon cigarette smoke exposure.

## Discussion

This study represents the first comprehensive investigation employing a broad multi-omics approach to elucidate the biological responses of airway epithelial cells to chronic smoke exposure. We delineated the transcriptional and epigenetic profile of the airway epithelium upon cigarette smoke exposure. Furthermore, we demonstrated a robust association between epigenetic regulation and the transcriptional response of oxidative stress and detoxification responses to this exposure. Cigarette smoke induced epigenetic marks to regulate gene expression, being positioned within smoking-related TF binding sites. The upregulated genes, particularly *ALDH3A1*, showed protective effects against cigarette smoke exposure in lung epithelial cells, as loss of function of these genes exacerbated the loss of epithelial integrity and increased epithelial cell death upon cigarette smoke exposure. These genes were predominantly expressed by ciliated and goblet cells, indicating that the oxidative stress and detoxification responses are localized to the surface layer of the airway wall.

Previous work has shown that cigarette smoking induces epigenetic changes in the small airway epithelium, accompanied by altered gene expression profiles.^21^ Our study builds upon and extends these findings by integrating methylation, transcriptomic, and proteomic data derived from the NORM asymptomatic cohort. This multi-omics strategy enabled us to not only confirm key epigenetic alterations but also map them to TF binding sites and link them directly to functional outcomes *in vitro* using CRISPR-Cas9-mediated gene KO. Thus, our work provides a more mechanistic and functionally validated understanding of how epigenetic regulation mediates the airway epithelial response to chronic cigarette smoke exposure.

Our findings regarding the higher expression of genes related to oxidative stress and detoxification pathways upon chronic smoke exposure are consistent with previous studies.^22^^,^^23^ Furthermore, these genes are likely under the direct regulation of the TFs NRF2 and AhR of which, the binding sites are epigenetically regulated. Both NRF2 and AhR play significant roles in cellular responses to environmental pollutants.^24^^,^^25^ Mice deficient in AhR display more severe pulmonary inflammation upon smoking,^26^ while mice deficient in NRF2 are highly susceptible to cigarette smoke-induced emphysema,^27^ providing evidence that both TFs have essential roles in regulating genes that protect against cigarette smoke. Our enrichment analysis of smoking-induced methylation sites support the hypothesis that the expression of detoxification and antioxidant genes is tightly regulated, and that their upregulation in response to prolonged smoke exposure is likely a result of hypomethylation of these sites.

Comparative studies showed that the lungs of humans exhibit higher expression of genes associated with the response to smoke compared to other primates, likely due to our historical practice of cooking food over open flames, resulting in direct exposure to associated smoke.^28^ Future studies should be performed to further elucidate the evolutionary protective mechanisms humans developed in response to the invention of fire and cooking. Studies may be performed comparing subjects that regularly perform indoor cooking without proper ventilation to subjects that do have proper ventilation. Additionally, our results should be compared to studies investigating the gene expression responses to other harmful exposures such as, air pollution, microplastics or industrial waste exposures. NQO1 is considered to have an antioxidant role during smoke exposure through the reduction of endogenous quinones, such as *p*-benzosemiquinone, a prominent constituent of cigarette smoke. In contrast, ALDH3A1 acts as an oxidant to catalyze the oxidation of reactive aldehydes in cigarette smoke.^29^ In our study, knocking out ALDH3A1 led to reduced epithelial barrier function and cell survival. Supporting these results, a previous study showed that suppression using small interfering RNA (siRNA) targeting ALDH3A1 or AhR decreased the survival of human bronchial epithelial cells during smoke exposure.^29^^,^^30^ Our study revealed that ADLH3A1 and NQO1 are specifically expressed in differentiated epithelial cells localized to the lumen, indicating that the oxidative stress response in the airways is confined to cells on the airway luminal surface directly exposed to inhaled smoke. In cell culture, our findings and those of others have shown that these genes are upregulated in undifferentiated basal cells in response to CSE, indicating that the lack of basal cell responses to smoke exposure is not due to an inability to respond but likely due to a lack of direct exposure. This has important implications on the validity of most smoking models, which directly expose cells found deeper in the airways, such as basal cells.

Overall, our study uses a multi-omics approach to identify and validate the cellular response of the lung epithelium to cigarette smoking. Here, we showed that the airways are epigenetically regulated to promote the expression of oxidative stress-response genes, thereby mitigating the effects of chronic smoke exposure. Importantly, this response is specific to airway surface epithelial cells and does not extend to the basal cell layer.

### Limitations of the study

Our study is limited by that fact that only the effect of cigarette smoking was investigated, omitting the effects of other exposures contributing to chronic respiratory diseases, such as air pollution, exhaust fumes, and microplastics. Furthermore, despite having used several omics layers, no proteomic datasets were used to validate the gene expression data.

## Resource availability

### Lead contact

Requests for further information and resources should be directed to and will be fulfilled by the lead contact, Dr. Simon D. Pouwels (s.d.pouwels@umcg.nl).

### Materials availability

All data are available in the main text or the supplementary materials. Raw data as can be obtained from the authors upon reasonable request.

### Data and code availability

• Data reported in this paper will be shared by the lead contact upon request.
• All code used for data processing, analysis, and figure generation in this study have been deposited at Github and are publicly available as of the date of publication. Accession numbers are listed in the key resources table.
• Any additional information required to reanalyze the data reported in this paper are available from the lead contact upon request.

## Acknowledgments

This research did not receive any specific grant from funding agencies in the public, commercial, or not-for-profit sectors.

## Author contributions

Conception and design of the study, S.D.P., A.F., V.G., M.v.d.B., and I.H.H.; acquisition of data: S.D.P., A.F., S.N.H.R., A.C.Y.Y., T.J.L., and A.L.; analysis of data, S.D.P., A.F., V.G., H.C., and R.M.M.; revising manuscript, S.D.P., C.-A.B., T.J.L., V.G., A.F., I.H.H., M.v.d.B., and H.C.

## Declaration of interests

The authors have no competing interests.

## Declaration of generative AI and AI-assisted technologies in the writing process

No AI or AI-assisted tools or technologies were used for the preparation of this manuscript.

## STAR★Methods

### Key resources table

**Table:** 

REAGENT or RESOURCE	SOURCE	IDENTIFIER
**Biological samples**		

Healthy controls	University Medical Center Groningen	NORM cohort (NCT00848406)

**Chemicals, peptides, and recombinant proteins**		

Annexin-V FITC	Immunotools	Cat. No. 31490013
Propidium Iodide	Sigma-Aldrich	Cat. No. 81845

**Experimental models: Cell lines**		

A549 cells	ATCC	RRID:CVCL_0023

**Software and algorithms**		

All code used for data processing, analysis, and figure generation in this study.	Github	https://github.com/UTS-Bioinformatics/Paper_Smoking_Multi_Omics
R (4.1.2. or higher)	R Core Team	https://cran.r-project.org/
Limma	R package	https://bioinf.wehi.edu.au/limma/
SAMtools	Samtools	V.1.2
FastQC tool	FastQC	V0.11.3
Benchling	Benchling	V2018

### Experimental model and study participant details

The Study to Obtain Normal Values of Inflammatory Variables From Healthy Subjects (NORM; ClinicalTrials accession no.: NCT00848406) includes 37 asymptomatic smokers and 40 never-smokers, defined by the absence of respiratory symptoms, normal lung function of FEV_1_/forced vital capacity (FVC) >70% and FEV_1_ >80% predicted, and no bronchial hyperresponsiveness to methacholine. Bronchial biopsies were obtained for comprehensive analysis of whole-transcriptome expression and methylation patterns (NCBI-GEO: GSE237252) using RNA sequencing and 450K methylation arrays, respectively. All study protocols were approved by the medical ethic committee of the University Medical Center Groningen (UMCG), The Netherlands and all subjects provided written informed consent. The clinical characteristics of the study participants have been published previously.^1^ In short, the average age is 36 years, 47% is female, and the average FEV_1_ percent predicted is 108%.

For *in vitro* experiments human lung adenocarcinoma A549 cells were used (CCL-185, ATCC). Cells were verified to be mycoplasma negative on a monthly basis.

### Method details

#### Methylation analysis

The methylation analysis, including the processing and quality control of the Infinium Human Methylation 450K BeadChip has been previously described.^31^ In short, DNA samples were purified by precipitation and rinsed with ethanol. Next, sodium bisulfite was used to convert unmethylated cytosine bases into uracil, followed by a whole genome amplification step, and hybridization to the Infinium HumanMethylation450 BeadChip array. All beadchips were analyzed with a blood DNA sample from a single female as control. Differential methylation analysis was conducted between current (n=33) and never smokers (n=36) using the R package limma,^32^ adjusting for age, sex and principle component analysis based on technical probes.^33^ An Benjamini–Hochberg correction for multiple testing was applied and an adjusted p-value of <0.05 was considered statistically significant.

#### Gene expression analyses

Gene expression analysis was performed on all 77 subjects of the NORM cohort. Bronchial biopsies were taken from segmental divisions of the main bronchi, 3rd-6th generation. Biopsies frozen in Tissue-Tek (VWR, Radnor, PA) at −80°C were thawed at room temperature and cut from the blocks when they were semi-solid. Total RNA was extracted using AllPrep DNA/RNA Mini kits (Qiagen, Venlo, the Netherlands). Samples were lysed in 600 μL RLT-plus buffer using an IKA Ultra Turrax T10 Homogenizer, and RNA was purified according to the manufacturer’s instructions. RNA samples were dissolved in 30 μL RNAse free water. Concentrations and quality of RNA were checked using a Nanodrop-1000 and run on a Labchip GX (PerkinElmer, Waltham, MA). RNA samples were further processed using TruSeq Stranded Total RNA Sample Preparation Kits (Illumina, San Diego, CA), using an automated procedure in a Caliper Sciclone NGS Workstation (PerkinElmer, Waltham, MA). All cytoplasmic and mitochondria rRNA was removed (RiboZero Gold kits). The obtained cDNA fragment libraries were loaded in pools of multiple samples unto an Illumina HiSeq2500 sequencer using default parameters for paired-end sequencing (2×100 bp).

Next, the trimmed fastQ files were aligned to build b37 of the human reference genome using HISAT (v0.1.5), allowing for two mismatches. Before gene quantification, SAMtools (v1.2) was used to sort the aligned reads. Gene-level quantification was performed by HTSeq (v0.6.1p1) using Ensembl v75 as gene annotation database. QC metrics were calculated for the raw sequencing data, using the FastQC tool (v0.11.3). Alignments of 77 subjects were obtained. QC metrics were calculated for the aligned reads using Picard-tools (v1.130) (http://picard.sourceforge.net), CollectRnaSeqMetrics, MarkDuplicates, CollectInsertSize-Metrics and SAMtools flagstat. In addition, the concordance between sex-linked (XIST and Y-chromosomal genes) gene expression and reported sex was checked. All samples were concordant. This resulted in high-quality RNA-seq data from 77 subjects.^1^

Additionally, gene expression data was used from two publicly available microarray datasets of healthy airway epithelial cells cultured at Air-Liquid Interface (ALI) that were treated with whole cigarette smoke (NCBI-GEO: GSE30660,^34^ *n* = 3; and NCBI-GEO: GSE82137,^35^ *n* = 4). ALIs in the GSE30660 dataset were exposed for 30 min on four separate days to whole cigarette smoke (*n* = 4) compared to air exposure. Those from the GSE82137 dataset were treated with a 48-min exposure on day one with whole cigarette smoke and then rested for 24 h, compared to air exposure. Microarray analysis was conducted using R software v3.02, using limma package, and normalized using robust multi-array average. A paired linear analysis was conducted using limma comparing treatment and control.

#### Comparing gene expression between humans and primates

For the comparison of gene expression profiles between human and non-human primate lung tissues, publicly available RNA-seq data from healthy lung tissue of Korean non-smoker females (*n* = 6) (NCBI-GEO: GSE37765) and non-human primates included in the Non-human Primate Reference Transcriptome Resource (NHPRTR)^16^ was used, which includes Baboon, Chimpanzee, Rhesus macaque, Cynomolgus macaque, Squirrel monkey, Sooty mangabey, Pig-tailed macaque, Marmoset and Mouse Lemur. The quality of all raw RNA-seq data was checked using FastQC. Reads were mapped against the primary human genome assembly (GRCh38) and quantified with STAR aligner (v2.4.2a) using release 83 of the human gene annotation from Ensembl (http://www.ensembl.org). Differentially expressed genes were selected from a list of 232 smoking-related genes requiring an average expression level of at least 1 CPM (*n* = 176 genes). The normalized CPM expression values were z-transformed before clustering and plotting was done using the pheatmap package.

#### Transcription factor enrichment and ChIP-Seq analyses

The enrichment analysis for NRF2 and AhR was conducted using GSEA on the following publicly available databases NCBI-GEO: GSE38332^10^ and NCBI-GEO: GSE109576,^11^ respectively. GSE38332 investigated the effects of NRF2 inhibition on A549 cell line using siRNA; control A549 cells (*n* = 3) compared to NRF2 siRNA A549 cells (*n* = 3), while GSE109576 investigated the effects of AhR inhibition on A549 expression with 4 h of treatment with CH-223191 (a selective inhibitor of AHR); control A549 cells (*n* = 3) compared to A549 cells + CH-223191 (*n* = 3). ChIP-Seq analysis was conducted on two publicly available datasets during the activation of NRF2 (NCBI-GEO: GSE75812^36^) and AhR,(NCBI-GEO: GSE90550^17^) respectively. ChIP-Seq for AhR was analyzed in the human breast cancer epithelial cell line MCF-7 in the presence and absence of treatment with TCDD, an activator of AhR, for 24 h (GSE90550). ChIP-Seq for NRF2 was analyzed in the airway epithelial cell line BEAS-2B in the presence and absence of treatment with the dietary isothiocyanate, sulforaphane (SFN), an activator of NRF2 (GSE75812). Both ChIP-seq datasets were used to identify generalizable AhR or NRF2 DNA-binding sites, as ligand-dependent regulatory mechanisms are conserved across epithelial cell types.^37^^,^^38^

#### Spatial transcriptomics analysis

To investigate the specificity of the genes associated with smoking, the gene signature derived from all significantly upregulated genes in smokers compared to non-smokers was applied to a publicly available spot-based spatial transcriptomics dataset of healthy human lungs (ArrayExpress: E-MTAB-11640)^12^ obtained through the 10x Visium platform. Our analysis focused specifically on samples extracted from the parenchyma-enriched region of the lungs, comprising data from age- and sex-matched asymptomatic current smoker (*n* = 1) and never smoker (*n* = 1) (Table S1), as these were the only samples available in the dataset relevant to our study. Airway club cell marker genes (SCGB1A1 and SCGB3A1) and visualization of hematoxylin and eosin (H&E)-stained lung tissue sections were employed to identify and locate the small airways within the lung parenchyma region.

#### Single-cell RNA sequencing analysis

To investigate which cell types express genes that change upon smoke exposure the following cell types (Club 1, Basal 1, Goblet 1, Basal 2, Ciliated 1, Club 2, Goblet 2, Club 3, Ciliated 2) were analyzed from single cell RNA-sequencing data from current (*n* = 6) and never smokers (*n* = 6) (NCBI-GEO: GSE131391).^13^ In a second analysis the following cell types were merged, basal, club, goblet, and ciliated cells. A signature was created of the genes that displayed higher expression in current compared to never smokers from the RNA-sequencing data (FC > 1, FDR<0.05), and genes that were more highly expressed in the lungs of humans compared to other primates. UMAPS and violin plots were created based on smoking status.

#### CRISPR knockout cells

Guide RNA (gRNA) was designed using the online web tool Benchling (v2018). A single gRNA was created for each of the four different genes (ALDH3A1, CYP1A1, CYP1B1, NQO1), targeting a common exon contained in all known splice variants determined by Ensembl. The CRISPR-Cas9 construct was transfected into A549 cells seeded at 100,000 cells/well in growth medium (RPMI 1640 medium, Lonza, Basel, Switzerland) supplemented with 10% Fetal Bovine Serum (FBS, Sigma-Aldrich, St. Louis, USA) and 1% Penicillin/Streptomycin (P/S, Gibco, California, USA) in 12 well plates (Sigma-Aldrich). The transfection of the px458 CRISPR-Cas9 construct was performed with Lipofectamine 3000 Transfection Reagent (ThermoFisher Scientific) according to the manufacturer’s protocol.

After 24 h of incubation, transfected cells were harvested and sorted based on GFP using a single cell sorter (SH800S Cell Sorter; Sony Biotechnology, Weybridge, UK) into a 96 well plate. To validate the knockout of selected genes, DNA was extracted using QIAamp DNA Investigator Kits (Ref. No. 56504; QIAGEN) according to the manufacturer’s protocol. Regions surrounding the cut site were amplified by PCR and the samples were sequenced at BaseClear (Leiden, Netherlands), and subsequently assessed with ICE and Benchling. The KO was confirmed by Sanger sequencing (Figure S1).

In order to prepare cigarette smoke extract (CSE) two 3R4F research cigarettes without filters (Tobacco Research & Development Center, Lexington, KY) were bubbled through 25 mL of RPMI 1640 medium (Lonza) supplemented with 1% P/S (Gibco), using a high flow peristaltic pump (Watson Marlow 603S, Rotterdam, The Netherlands). The obtained solution was used as 100% CSE. The extract was prepared freshly for each experiment and used within 30 min.

#### Electric cell-substrate impedance sensing

Electrical resistance was recorded in real-time using the ECIS Z Theta system (Applied Biophysics) with associated software. 8W10E + PET electrode chamber arrays were equilibrated with serum-free growth medium for 2 h. A549 cells were seeded in duplicates at 25,000 cells/well. After serum-starvation for 24 h the cells were exposed to medium or 20% CSE for 24 h. Resistance was measured from 0 to 72 h at a low-frequency resistance of 40 Hz, to assess cell-cell contacts.

#### Annexin-V/PI staining

A549 cells were treated with 0%, 50% and 100% CSE for 4 h. Afterward, cells were incubated with fresh serum-free medium for 20 h. Cells were collected and washed twice using Cell Staining Buffer (BioLegend; Cat. No. 420201) and subsequently centrifuged (1,200g, 5 min). Cells were resuspended and stained with 40:42 Annexin-V Binding Buffer (BioLegend; Cat. No. 422201), 1:42 Annexin-V FITC (ImmunoTools; Cat. No. 31490013) and 1:42 Propidium Iodide [1.0 mg/mL], and incubated for 15 min (RT, protected from light). Fluorescence was assessed using a FACSCalibur (Becton-Dickinson Medical Systems, Heidelberg, Germany).

### Quantification and statistical analysis

Differential gene expression and methylation analyses were performed using linear modeling with empirical Bayes moderation as implemented in the limma package. Multiple testing correction was applied using the Benjamini–Hochberg FDR, with adjusted *p* < 0.05 considered statistically significant. GSVA enrichment scores were compared between groups using unpaired t-tests. All data are presented as median ± interquartile range with individual datapoints plotted on top.
