# Supplementary material for: A comprehensive multi-omics and functional study of evolutionary adaptive responses to smoke
Source: iScience. 2026 Apr 1;29(5):115547. doi: 10.1016/j.isci.2026.115547 (PMC13092862; doi:10.1016/j.isci.2026.115547)
Supplement: Document S1. Figure S1, Tables S1, and S3 [file mmc1.pdf]

iScience, Volume *29*

Supplemental information

A comprehensive multi-omics and functional study of evolutionary adaptive responses to smoke

Simon D. Pouwels, Hao Chen, Senani N.H. Rathnayake, Andy Lan, Rashad M. Mahbub, Anna Chi Ying Yeung, Corry-Anke Brandsma, Thamar J. Lobo, Victor Guryev, Irene H. Heijink, Maarten van den Berge, and Alen Faiz

Supplementary data:

Supplementary Figure 1:

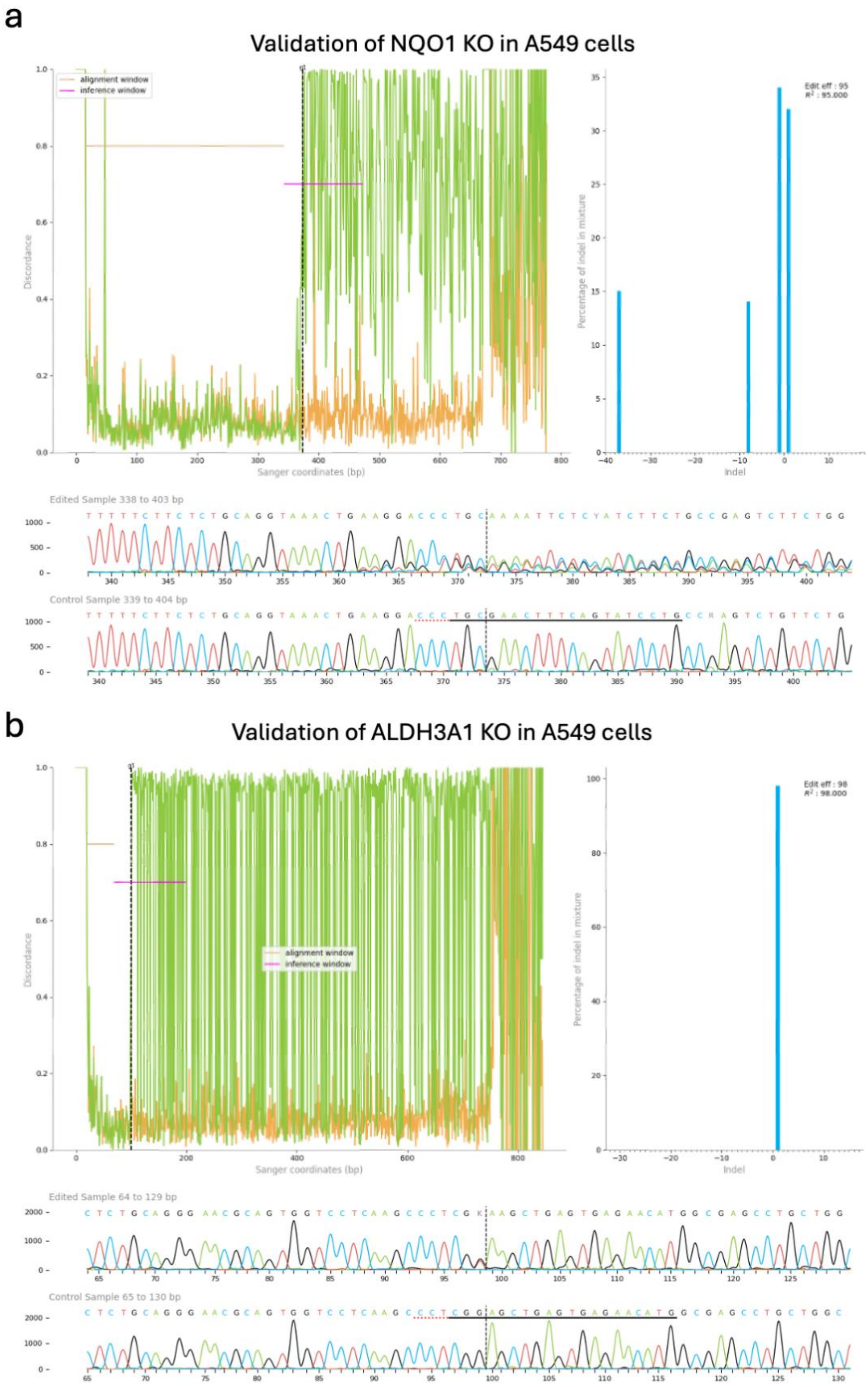

Supplementary Figure 1: Sanger sequencing knock-out results for A) *NQO1* and B) *ALDH3A1* in A549 cells.

Table S1. Visium spatial transcriptomics sample characteristics

Sequencing ID in original publication	Tissue region from H&E staining	Donor	Age	Smoking status	Years of smoking	Sex	Permeabilisation time for Visium processing	Protocol
WSA_LngSP10193347	Parenchyma	A37	55-60	Current smoker	30	F	24min	standard
WSA_LngSP9258469	Parenchyma	A47	55-60	Never smoker	NA	F	24min	standard

Table S3. KEGG pathway analysis of smoking signature

Pathway	Adjusted p value
Steroid hormone biosynthesis	0.000145
Mucin type O-glycan biosynthesis	4.44e-06
Retinol metabolism	0.00693
Metabolism of xenobiotics by cytochrome P450	0.00036
Metabolic pathways	5.47e-05
Chemical carcinogenesis	0.000645

Log2 fold change >2 or <-2, adjusted p value <0.05.
